# Association between social determinants of health and pediatric traumatic brain injury outcomes

**DOI:** 10.3389/fneur.2024.1339255

**Published:** 2024-03-14

**Authors:** Kendall Parsons, Makda G. Mulugeta, Gabrielle Bailey, Scott Gillespie, Laura M. Johnson, Hannah E. Myers, Andrew Reisner, Laura S. Blackwell

**Affiliations:** ^1^Children's Healthcare of Atlanta, Atlanta, GA, United States; ^2^Department of Pediatrics, Emory University School of Medicine, Emory University, Atlanta, GA, United States; ^3^Medical College of Georgia, Augusta, GA, United States; ^4^Department of Neurosurgery, Emory University School of Medicine, Emory University, Atlanta, GA, United States

**Keywords:** pediatric, traumatic brain injury, social determinants of health, race, outcomes, mortality

## Abstract

**Introduction:**

Social determinants of health (SDH) are factors that may impact outcomes following pediatric traumatic brain injuries (TBI). The purpose of this study was to investigate the relationship between race and functional outcomes in a diverse pediatric population. We further explored how this association may be modified by SDH factors, including insurance status, social vulnerability, and child opportunity.

**Methods:**

A cohort study (*N* = 401) of children aged 0–18 [median = 9.22 years (IQR: 3.56–13.59)] presenting to the Emergency Department at Level I and II Trauma Centers with mild to severe head injuries. Geocoded variables were used to evaluate SDH. The sample was described overall and by racial/ethnic group, which were adjusted for confounders using inverse propensity treatment weights (IPTW). Weighted and unweighted Firth logistic regression models (mortality) and generalized linear regression models (GOS-E scores) were reported without and then with potential effect modifiers.

**Results:**

The sample is majority male (65.84%); race/ethnicity are as follows: White (52.37%), Black/African Americans (35.91%), and Hispanic (11.72%). Black (31.25%) and Hispanic (27.66%) patients had higher rates of severe TBI. 35.89% of White patients were categorized as more socially vulnerable compared to 62.68% Black and 70.21% Hispanic patients. A total 63.64% of White patients were from higher opportunity neighborhoods, compared to 25.87% of Black and 51.06% of Hispanic patients. A total 50.95% of White patients, 25.87% of Black patients, and 17.02% of Hispanic patients were privately insured. There were no differences found between racial and ethnic groups on mortality or GOS-E scores.

**Discussion:**

Patients from minority backgrounds had more severe injuries, many resulting from pedestrian vs. motor vehicle accidents. Additionally, patients from minority backgrounds experience more social vulnerability and lower opportunity. Despite these discrepancies, we did not observe differences on rates of mortality or functional outcomes in either racial or ethnic groups. SDH were not found to impact outcomes. Further research is needed to determine how these complex social and environmental variables impact health outcomes.

## 1 Introduction

Traumatic brain injury (TBI) is a leading cause of morbidity and mortality in the United States. The pediatric population is uniquely vulnerable, with over 50,000 annual hospitalized cases (1). Despite an overall decrease in pediatric TBI mortality due to enhanced emergency access and the introduction of evidence-based guidelines (2), the longer-term cognitive, psychological, social, and adaptive morbidities persist among survivors. Pediatric TBI is also heterogeneous, given the wide range of possible mechanisms of injury, severity of injury, and the factors that affect the secondary responses to TBI (3, 4). However, only recently has research examined specific social and environmental factors that may play a role in TBI incidence, management, and outcomes (5).

The influence of social and environmental factors, termed social determinants of health (SDH), on health inequities is substantial, particularly in TBI. Prior studies have shown that TBI disproportionately affects patients from lower socioeconomic backgrounds and minority races (6–10). A recent national study found that racial minorities (Black, Hispanic, and Native Americans), females, older children, and children in lower socioeconomic groups were at increased risk of poor outcomes following TBI, including longer length of stay in the hospital, increased medical complications, higher rates of mortality, and worse functional outcomes (10). Additional contributing factors include decreased use of protective devices (e.g., helmets and restraining seat belts), less access to trauma centers, and underestimated triage scores for minority patients (11–13). Additionally, lower education levels have been shown to contribute to misconceptions around head injuries as well as decreased reporting (12, 14). Caregivers of pediatric patients also report scheduling conflicts and lack of resources as barriers to follow up care with publicly insured and uninsured minority parents reporting lack of resources as the primary barrier (15). It is noteworthy that these results are not uniform, and there are still large gaps in our knowledge.

The term SDH is defined by the World Health Organization as “non-medical factors that influence health outcomes” and are impacted by socioeconomic status and structural mechanisms of society that create inequity (16, 17). These are modifiable and unmodifiable conditions that encompass five key domains: economic, education, social and community context, health and health care, and environment (5, 18). Given the number of variables that fit within SDH, several indices were developed to aggregate factors into holistic measures of life condition to support research within this area. Many of these indices use geocoding, through patient addresses and ZIP codes, which correlates with social, environmental, and demographic information found on public databases such as the U.S. Census.

In order to better understand the impact of neighborhood conditions, researchers developed the Child Opportunity Index (COI). Using data from sources such as the U.S. Census Bureau, Environmental Health Agency, and the Department of Education, the COI measures the quality of resources and conditions (e.g., good early childhood education centers and schools, green spaces, access to healthy food, low poverty) that allow children to develop healthily in the neighborhoods they live in (19). Many studies have found associations with COI and hospital outcomes such that lower-opportunity neighborhoods correlate with increased hospital re-admission or severity of injury (20–22). However, limited studies have utilized the COI within pediatric TBI (22).

Originally developed by the Centers for Disease Control and Prevention (CDC), the social vulnerability index (SVI) was created in order to guide the allocation of resources to communities in need during disasters or disease outbreak (23, 24). This tool organizes 15 census variables into themes of socioeconomic status, including household composition and disability, minority status and language, and housing type and transportation, in order to rank and characterize a community. While this measure has been used to measure areas of vulnerability within a community for health-related applications, it has yet to be applied to the TBI populations.

The aims of this study are to examine the association between patient race and functional outcomes, including mortality and functional outcomes, in pediatric TBIs. We hypothesized that there will be differences between racial groups, including higher rates of mortality and lower GOS-E peds scores, the gold standard in outcome measurements, in patients from minority backgrounds. Further, we expected that the relationship between race and TBI-related outcomes will be moderated by SDH variables (COI, Social Vulnerability Index, and insurance).

## 2 Materials and methods

### 2.1 Patient population

The current study is part of a larger prospective investigation examining outcomes in children and adolescents presenting to the Emergency Department at a Level I or a Level II trauma center with a TBI between March 2017 and June 2021 (25). Participants included children <18 years of age who were diagnosed on arrival with a TBI by attending physicians. Additional information on the study protocol can be found here (25). All inclusion and exclusion criteria remain the same except that only patients with the following racial categories were used, based on the primary aims of the study: White, Black, Hispanic. This study was approved by our institutional IRB.

### 2.2 Study variables

Demographic variables were obtained from caregivers or from electronic medical record in instances where caregivers were not available. Insurance status was obtained from electronic medical records based on status at the time of injury. Glasgow Coma Scale (GCS) was obtained from patient electronic medical records and the lowest reported GCS score, including at the scene and during admission, was used. Patients were categorized into severity groups as follows: mild TBI (GCS 13–15), mild-complicated TBI (GCS 13–15 + skull fractures/intracranial injury), moderate TBI (GCS 9–12), and severe TBI (GCS 3–8). Glasgow Outcome Score-Extended Pediatrics (GOS-E Peds) was obtained through semi-structured interviews on the phone with caregivers of patients only in the moderate/severe TBI groups 6 months (+/– 1 month) post-injury. The GOS-E peds was utilized for this study based on recommendations for its use the NINDS common data elements (26, 27). Patients were categorized into the following scores: 1 = death, 2 = vegetative state, 3 = lower severe disability, 4 = upper severe disability, 5 = lower moderate disability, 6 = upper moderate disability, 7 = lower good recovery, and 8 = upper good recovery.

### 2.3 Geocoding

The patient's home address linked to the hospitalization event was used for geocoding across two databases. The Child Opportunity Index (COI) was developed as a summary measure of the quality of neighborhoods in which children live across the US (28). The index quantifies 29 indicators of neighborhood conditions and resources that affect children's healthy development. Each indicator is transformed to a z-score, standardized, and weighted by how strongly it predicts children's long-term health and economic outcomes. Indicators are then combined into overall and domain scores (education, health and environment, and social and economic), and divided into nationally normed quintiles. For the purpose of our study, we used the nationally normed COI overall z-scores. Higher values indicate more opportunity.

The Social Vulnerability Index (SVI) was developed and validated by the CDC/Agency for Toxic Substances and Disease Registry (ATSDR) (23). The index is derived from 15 US census tract variables from the American Community Survey data, and groups them into 4 domains: socioeconomic status, household composition and disability, minority status and language, and housing type and transportation. For each Census tract, there are generated percentile ranks, ranging from 0 (lowest vulnerability) to 1 (highest vulnerability) for all 15 variables combined. In this dataset, the social vulnerability index ranges from 0.0009 to 0.9661 and was dichotomized into patients with more vulnerability or less vulnerability based on whether patients were above or below the average social vulnerability for Georgia (0.4999).

### 2.4 Statistical analyses

Data were analyzed using SAS v.9.4 (Cary, NC) and CRAN R v.4.3 (Vienna, Austria), and 0.05 was used as the threshold for assessing statistical significance throughout. Analysis took place in several stages. First, descriptive statistics were calculated for the sample overall and compared by racial/ethnic groups. Second, racial/ethnic groups were balanced by potential demographic and clinical confounders (age, sex, severity of injury) using inverse propensity treatment weights (IPTW). Weights were derived from the *twang* v.2.5 package in CRAN R and confounders were considered balanced when standardized mean differences (SMD) <0.25. Average treatment effect (ATE) weights were calculated using 10,000 trees in a gradient boosted model (GBM), and interaction depth specified at 3, and a stop method based on mean effect size. The final weights were trimmed at the 1% and 99% and stabilized to approximately match the original study sample size. Third, clinical outcomes analysis considered the association between race with mortality (binary outcome) and GOS-E Peds (continuous outcome). For each outcome, three factors were considered as potential effect modifiers: social vulnerability index, childhood opportunity index, and insurance status. When mortality was the outcome, Firth's Penalized Likelihood was utilized to estimate odds-ratios and confidence intervals, which adjusts estimates to account for the bias that can occur with rare binary outcomes. For GOS-E Peds, general linear regression models were used, and results presented with least-squares means, 95% CI, and *p*-values. All models present unweighted and weighted results.

## 3 Results

### 3.1 Patient demographics

The final sample included 401 patients, with the majority male (65.84%) and sustaining mild TBIs (65.38%). Patients were predominantly school age (median = 9.22 years), with an interquartile range of age between 3.56 to 13.59 years. Of the patient racial distribution, 35.91% were Black, 52.37% were White, and 11.72% were Hispanic. Majority of patients sustained their injury due to a fall, followed by motor vehicle collision and struck by an object. Overall, there were low rates of mortality (3.99%) in the sample and 14.46% of patients required intensive inpatient rehabilitation services (Table 1).

**Table 1:** Outcomes and social determinants of health effect modifiers by race/ethnicity.

	**Overall (*N =* 401)**	**White (*N =* 210)**	**Black (*N =* 144)**	**Hispanic (*N =* 47)**	***P*- value**
**Outcomes**					
Died (Yes)	16 (3.99)	6 (2.86)	7 (4.86)	3 (6.38)	0.429
GOSE-peds (continuous, true score)	7 (4–8)	7 (5–8)	5 (2–8)	6 (1–8)	0.722
Positive neuroimaging findings					0.127
No	148 (37.0)	68 (34.35)	57 (42.54)	12 (27.27)	
Yes	252 (63.0)	130 (65.65)	77 (57.46)	32 (72.73)	
Hospital days (Median/IQR)	2 (1–5)	1 (0–4)	2 (1–11.5)	2 (1–6)	**0.002**
Neurosurgery					0.1 03
No	379 (89.18)	193 (91.90)	122 (84.72)	41 (87.23)	
Yes	46 (10.82)	17 (8.10)	22 (15.28)	6 (12.77)	
Mechanism of Injury					**<0.001**
Fall	128 (31.92)	76 (36.19)	37 (25.69)	15 (31.91)	
Motor vehicle crash	108 (26.93)	45 (21.43)	50 (34.72)	13 (27.66)	
Pedestrian vs. motor vehicle	38 (9.48)	14 (6.67)	19 (13.19)	5 (10.64)	
Bike	17 (4.24)	10 (4.76)	4 (2.78)	3 (6.38)	
Struck by/against	39 (9.73)	26 (12.38)	7 (4.86)	6 (12.77)	
Confirmed abuse	7 (1.75)	2 (0.95)	4 (2.78)	1 (2.13)	
Suspected abuse	23 (5.74)	8 (3.81)	13 (9.03)	2 (4.26)	
ATV	31 (7.73)	26 (12.38)	4 (2.78)	1 (2.13)	
Other	10 (2.49)	3 (1.43)	6 (4.17)	1 (2.13)	
Rehab					**0.001**
No	343 (85.54)	192 (91.43)	112 (77.78)	39 (82.98)	
Yes	58 (14.46)	18 (8.57)	32 (22.22)	8 (17.02)	
**Effect Modifiers**					
Social Vulnerability Index					**<0.001**
Less vulnerable	201 (50.50)	134 (64.11)	53 (37.32)	14 (29.79)	
More vulnerable	197 (49.50)	75 (35.89)	89 (62.68)	33 (70.21)	
Child Opportunity Index					**<0.001**
Less opportunity	205 (51.38)	76 (36.36)	106 (74.13)	23 (48.94)	
More opportunity	194 (48.62)	133 (63.64)	37 (25.87)	24 (51.06)	
Insurance					**<0.001**
None	61 (15.25)	28 (13.33)	27 (18.88)	6 (12.77)	
Private	152 (38.00)	107 (50.95)	37 (25.87)	8 (17.02)	
Public	187 (46.75)	75 (35.71)	79 (55.24)	33 (70.21)	

*Statistics shown are Median (IQR); n (%)*. Bold means statistically significant *p* <0.01.

There were no differences in age or sex across racial groups (Table 2). There was a statistically significant association between severity of injury and race (*p* <0.001). Black and Hispanic patients had higher rates of severe TBI compared to White patients (Table 1), with Blacks experiencing severe TBI at twice the rate of Whites. Similarly, a larger percentage of Black patients sustained moderate TBIs, compared to both Hispanic and White patients. However, nearly half of White patients sustained mild-complicated TBIs, compared to smaller portions of Hispanic and Black patients. Mild TBIs were sustained at similar rates across racial groups. With regard to mechanism of injury, differences across racial groups were also observed (*p* <0.001; Table 1), such that Black and Hispanic patients were more likely to be injured in motor vehicle collisions, compared to White patients. Additionally, less White patients suffered injuries from pedestrian vs. car accidents, in contrast to Black and Hispanic patients. Black and Hispanic patients had similar percentages of confirmed abuse cases, double that of White patients. However, Black patients had more suspected abuse cases compared to Hispanic and White patients. More White patients were injured by all-terrain vehicles (ATVs) than Black and Hispanic patients.

**Table 2:** Balancing racial/ethnic groups to account for potential confounds (*N* = 401).

	**White (*N =* 210)**	**Black (*N =* 144)**	**Hispanic (*N =* 47)**	***P*- value**	**SMD**	**IPTW SMD**
**Confounding variables (to be balanced)**						
Child age (Median (IQR))	9.96 (3.61–14.34)	8.06 (3.38–12.47)	10.63 (4.34–13.82)	0.110	0.141	0.056
Sex				0.603	0.109	0.139
Male	136 (64.76)	94 (65.28)	34 (72.34)			
Female	74 (35.24)	50 (34.72)	13 (27.66)			
Severity of Injury				**<0.001**	0.403	0.081
Mild TBI	75 (35.71)	47 (32.64)	13 (27.66)			
Mild-complicated TBI	88 (41.90)	31 (21.53)	15 (31.91)			
Moderate TBI	18 (8.57)	21 (14.58)	6 (12.77)			
Severe TBI	29 (13.81)	45 (31.25)	13 (27.66)			

*Racial/Ethnic groups are balanced by age, sex, and severity of injury. SMDs*<*0.25 are considered balanced. IPTW SMDs are calculated using stabilized ATE IPTW, truncated at 1% and 99%*. Bold means statistically significant *p* <0.01.

On average, Black and Hispanic patients stayed in the hospital a day longer than White patients (*p* = 0.002). Although Hispanic patients had the highest mortality rate (6.38%) followed by Black patients (4.86%) and then White patients (2.86%), these differences were not statistically significant. Black and Hispanic patients had higher admission rates to inpatient rehabilitation compared to White patients (Table 1).

### 3.2 Race/ethnicity and differences in SDH

The SVI groups differed by racial/ethnic group (*p* <0.001, Table 1), such that lower percentages of White patients were categorized as “more vulnerable” in comparison to higher rates of Black and Hispanic patients. Similarly, there were significant differences between racial groups and childhood opportunity (*p* <0.001). Approximately two-thirds of White patients were in neighborhoods with “more opportunity” followed by Hispanic and then Black patients. With regard to insurance, almost half of our overall sample had public insurance, with significant differences noted between racial groups, including Hispanic patients having the highest rates, followed by Black and White patients, respectively.

### 3.3 Mortality by race/ethnicity and SDH

The mortality rate in the overall sample was 3.99%, which differed across racial groups: Hispanic patients (6.38%), Black patients (4.86%), and White patients (2.86%). Table 3 presents models that investigate the association of race/ethnicity and mortality, testing for three possible effect modifiers (COI, SVI, and insurance). Despite differences in overall mortality rates, there were no significant associations detected between racial/ethnic groups and mortality, and there was no evidence of effect modification for any of the three tested effect modifiers between weighted and unweighted models. For the sake of brevity, regression models will be reported. The following analysis will be descriptive, as small sample sizes in these groups limited the findings.

**Table 3:** Unweighted and weighted regression^†^ models, outcome: mortality *N* = 401.

**Characteristic**	**Alive, *N =* 385 Raw N (row%)**	**Deceased, *N =* 16 Raw N (row%)**	**Unweighted OR (95% CI)**	**P-Value**	**IPTW OR (95% CI)**	**P-Value**
**Race/Ethnicity**						
Black	137 (95.14)	7 (4.86)	Reference	-	Reference	-
White	204 (97.14)	6 (2.86)	0.58 (0.20, 1.71)	0.325	1.05 (0.27, 4.07)	0.948
Hispanic	44 (93.62)	3 (6.38)	1.44 (0.38, 5.41)	0.588	2.96 (0.25, 35.69)	0.394
**Social Vulnerability (Below Average), Lower Vulnerability** ***N** =* **201**						
**Race/Ethnicity**						
Black	50 (94.34)	3 (5.66)	Reference	-	Reference	-
White	130 (97.01)	4 (2.99)	0.50 (0.12, 2.11)	0.343	0.77 (0.13, 4.41)	0.766
Hispanic	13 (92.86)	1 (7.14)	1.60 (0.20, 12.58)	0.653	3.97 (0.13, 118.26)	0.426
**Social Vulnerability (Above Average), Higher Vulnerability** ***N** =* **197**						
**Race/Ethnicity**						
Black	85 (95.51)	4 (4.49)	Reference	-	Reference	-
White	73 (97.33)	2 (2.67)	0.65 (0.13, 3.16)	0.590	1.02 (0.14, 7.67)	0.981
Hispanic	31 (93.94)	2 (6.06)	1.51 (0.30, 7.6)	0.619	3.52 (0.17, 71.48)	0.412
**Childhood Opportunity Index (**<**Average), Lower Opportunity** ***N** =* **205**						
**Race/Ethnicity**						
Black	101 (95.28)	5 (4.72)	Reference	-	Reference	-
White	74 (97.37)	2 (2.63)	0.62 (0.13, 2.87)	0.54	1.02 (0.15, 7.04)	0.982
Hispanic	22 (95.65)	1 (4.35)	1.23 (0.19, 8.19)	0.83	4.17 (0.15, 113.52)	0.397
**Childhood Opportunity Index (**≥**Average), Higher Opportunity** ***N** =* **194**						
**Race/Ethnicity**						
Black	35 (94.59)	2 (5.41)	Reference	-	Reference	-
White	129 (96.99)	4 (3.01)	0.49 (0.10, 2.46)	0.389	0.66 (0.10, 4.46)	0.674
Hispanic	22 (91.67)	2 (8.33)	1.58 (0.25, 10.16)	0.631	2.40 (0.11, 51.06)	0.576
**Insurance (none)**, ***N** =* **61**						
**Race/Ethnicity**						
Black	24 (88.89)	3 (11.11)	Reference	-	Reference	-
White	27 (96.43)	1 (3.57)	0.38 (0.05, 2.89)	0.351	0.93 (0.08, 10.26)	0.953
Hispanic	6 (100)	0 (0)	0.54 (0.02, 14.74)	0.714	2.83 (0.03, 286.52)	0.659
**Insurance (Private)**, ***N** =* **152**						
**Race/Ethnicity**						
Black	37 (100)	0 (0)	0.4 (0.02, 8.19)	0.55	0.88 (0.04, 21.47)	0.938
White	104 (97.20)	3 (2.80)	Reference^*^	-	Reference	-
Hispanic	8 (100)	0 (0)	1.76 (0.07, 43.47)	0.731	7.15 (0.14, 380.02)	0.332
**Insurance (Public)**, ***N** =* **87**						
**Race/Ethnicity**						
Black	75 (94.94)	4 (5.06)	Reference	-	Reference	-
White	73 (97.33)	2 (2.67)	0.57 (0.12, 2.80)	0.489	1.32 (0.24, 7.31)	0.751
Hispanic	30 (90.91)	3 (9.09)	1.93 (0.44, 8.42)	0.384	4.18 (0.27, 65.11)	0.307

^**†**^*Firth's Penalized Likelihood has been applied to these regression models to account for the bias that can occur with rare outcomes*. ^*^Reference group changed to White, as no Black patients were in the deceased group for private insurance.

With regard to social vulnerability, White patients living in less vulnerable neighborhoods had a similar mortality rate to White patients living in more vulnerable neighborhoods (2.99% vs. 2.67%). Conversely, Black and Hispanic patients who lived in less vulnerable neighborhoods had higher mortality rates than those living in more vulnerable neighborhoods (5.66% and 7.14% vs. 4.49% and 6.06%, respectively). Regarding neighborhood opportunity levels, Black and White patients had similar mortality rates whether they came from lower opportunity neighborhoods (4.72% and 2.63%) or higher opportunity neighborhoods (5.41% and 3.01%). Hispanic patients from higher opportunity neighborhoods face twice the mortality rate that Hispanic patients in lower opportunity neighborhoods do (8.33% vs. 4.35%). In low opportunity neighborhoods, Hispanic patients had similar mortality rates to Black patients (4.35% vs. 4.72%). Mortality was highest in publicly insured patients, followed by uninsured patients, and privately insured patients. Across all insurance types, White patients had a consistent mortality rate of about 3%, while Black and Hispanic patients' rates varied greatly. Black patients who were uninsured had twice the mortality rate of Black patients who were publicly insured (11.11% vs. 5.06%).

### 3.4 Functional outcomes by SDH and race/ethnicity

A quarter of the sample (27.50%) had poor outcomes, as defined by the dichotomized GOS-E Peds score of 1–4. Hispanic patients had the highest average outcome score (95% CI 4.63–7.87), followed by White patients (95% CI 5.51–6.93) and Black patients (95% CI 4.73–6.42). Table 4 presents models that investigate the association of race/ethnicity and mortality and then tests three possible effect modifiers. Findings between weighted and unweighted models did not change interpretations of the results, and for the sake of brevity, will not be reported.

**Table 4:** Unweighted and weighted general linear regression models, outcome: GOS-E Peds (*N* = 80).

**Characteristic**	**Unweighted LS-Mean GOS-E peds true score (95% CI)**	***P*-value**	**IPTW LS-Mean GOSE true score (95% CI)^a^**	***P-*value**
**Race/Ethnicity**				
Black	4.94 (3.95, 5.93)	Ref	5.57 (4.73, 6.42)	Ref
White	6.10 (5.34, 6.85)	0.068	6.22 (5.51, 6.93)	0.250
Hispanic	5.27 (3.49, 7.05)	0.744	6.25 (4.63, 7.87)	0.462
**Social Vulnerability (Below Average), Lower Vulnerability** ***N** =* **41**				
**Race/Ethnicity**				
Black	4.92 (3.46, 6.39)	Ref	5.44 (4.16, 6.72)	Ref
White	6.21 (5.22, 7.19)	0.152	6.23 (5.41, 7.06)	0.300
Hispanic	1.00 (−4.29, 6.29)	0.159	1.00 (−8.89, 10.89)	0.379
**Social Vulnerability (Above Average), Higher Vulnerability** ***N** =* **39**				
**Race/Ethnicity**				
Black	4.95 (3.73, 6.16)	Ref	5.67 (4.61, 6.72)	Ref
White	5.85 (4.38, 7.31)	0.351	6.17 (4.94, 7.40)	0.537
Hispanic	5.70 (4.03, 7.37)	0.471	6.73 (3.75, 9.71)	0.507
**Childhood Opportunity Index (**<**Average), Lower Opportunity** ***N** =* **41**				
**Race/Ethnicity**				
Black	5.12 (4.04, 6.2)	Ref	5.65 (4.69, 6.6)	Ref
White	6.07 (4.67, 7.46)	0.288	6.38 (5.28, 7.48)	0.321
Hispanic	5.00 (2.59, 7.41)	0.928	6.18 (1.93, 10.43)	0.80
**Childhood Opportunity Index (**≥**Average), Higher Opportunity** ***N** =* **38**				
**Race/Ethnicity**				
Black	4.5 (2.3, 6.7)	Ref	5.57 (3.89, 7.25)	Ref
White	6.11 (5.07, 7.15)	0.192	6.11 (5.22, 7.00)	0.572
Hispanic	5.50 (3.30, 7.70)	0.525	6.31 (2.40, 10.22)	0.729
**Insurance (none)**, ***N** =* **7**				
**Race/Ethnicity**				
Black	4.14 (2.31, 5.98)	Ref	5.32 (3.69, 6.94)	Ref
White	2.50 (−0.93, 5.93)	0.403	2.80 (0.18, 5.42)	0.109
Hispanic	NA	NA	NA	NA
**Insurance (Private)**, ***N** =* **36**				
**Race/Ethnicity**				
Black	7.33 (5.72, 8.95)	Ref	7.41 (6.04, 8.77)	Ref
White	6.70 (5.77, 7.64)	0.504	7.04 (6.25, 7.82)	0.639
Hispanic	8.00 (3.15, 12.85)	0.796	8.00 (2.94, 13.06)	0.823
**Insurance (Public)**, ***N** =* **37**				
**Race/Ethnicity**				
Black	3.94 (2.72, 5.15)	Ref	4.68 (3.68, 5.68)	Ref
White	5.38 (4.04, 6.73)	0.116	5.31 (4.28, 6.35)	0.385
Hispanic	5.00 (3.47, 6.53)	0.283	5.67 (2.74, 8.59)	0.527

^a^ *IPTW weights are calculated using GBM with N* = *10,000 trees, stabilized and trimmed at 1% and 99%; Weights adjust for age, sex, and enrollment status as confounding covariates*

The observed outcome score gap between Black and White patients was larger in less vulnerable areas (5.44; 95% CI 4.16–6.72) vs. (6.23; 95% CI 5.41–7.06) compared to more vulnerable areas (5.67; 95% CI 4.61–6.72) vs. (6.17; 95% CI 4.94–7.40). Outcome scores were comparable between lower opportunity areas compared to higher opportunity areas across racial groups. White (6.38; 95% CI 5.28–7.48) and Hispanic (6.18; 95% CI 1.93–10.43) patients had higher GOS-E peds scores compared to Black patients (5.65; 95% CI 4.69–6.60) in lower opportunity areas. There were less differences across racial groups in the higher opportunity areas (Black: 5.57; 95% CI 3.89–7.25; White: 6.11; 95% CI 5.22–7.00; Hispanic: 6.31; 95% CI 2.40–10.22). Functional outcomes were highest for those with private insurance, with minimal differences across racial groups (Black: 7.41; 95% CI 6.04–8.77; White: 7.04; 95% CI 6.25–7.82; Hispanic: 8.00; 95% CI 2.94–13.06). Within uninsured patients (*N* = 7), Black patients (5.32; 95% CI 3.69–6.94) had higher outcome scores than White patients (2.80; 95% CI 0.18–5.42). Of the publicly insured patients, Black patients (4.68; 95% CI 3.68–5.68) had lower outcome scores than White (5.31; 95% CI 4.28–6.35) and Hispanic patients (5.67; 95% CI 2.74–8.59). Hispanic and Black patients who were privately insured scored 2–3 points higher in their 6-month TBI outcome follow-up than patients of the same race who were publicly insured.

## 4 Discussion

While racial disparities are well documented in the US health system, there has been less attention within the pediatric TBI literature, particularly how these disparities impact outcomes. The primary objective of this study was to better understand the social-environmental risk factors that contribute to outcomes in pediatric TBI. Our results revealed differences across racial groups with regard to TBI injury severity, mechanism of injury, and SDH. Despite these disaprities, we did not find group differences in mortality or functional disability.

Consistent with past literature, our study found differences between racial and ethnic groups on SDH variables, with both Black and Hispanic patients showing higher rates of social vulnerability and lower child opportunity ratings compared to Whites (29, 30). Despite these discrepancies, we were unable to detect differences in race/ethnicity and SDH variables on functional disability and mortality. One possible reason for this finding is that our mortality rates were quite low, leaving the groups within racial categories and SDH quite small, likely under powering our ability to see significant differences. It is also possible that these social and environmental factors may not be as strongly related to 6 months functional disability but are more impactful when examining patient functioning over years following their TBI. Additionally, we had a high attrition rate in our sample, leading to missing data in our functional disability score. When conducting follow up analyses on patients who did not complete the follow up visit, similar rates of missing were found between White and Black patients (~39%), whereas a higher rate was found among Hispanic patients (~50%).

Despite the GOS-E peds being considered a gold standard measure of global outcome following TBI, there are several limitations that are worth noting and may have contributed to our null findings. The GOS-E peds is a simple, practical index that rates patients on a crudely defined, ordinal scale, which makes it accessible and easy to use within prospective research studies (27, 31). However, the measure reflects functional deficits and disability from multiple causes, not exclusively brain injury (e.g., polytrauma), which makes it potentially insufficient at measuring the numerous specific sequelae of TBI. Prior studies in adults have found racial disparities on the GOS-E, however, these disparities have not been explored as strongly within pediatrics (32, 33). Given these limitations, it is possible that there are racial and ethnic disparities in sequelae of TBI in children that are not adequately being captured by GOS-E peds.

Geocoding variables are relatively new and have become increasingly popular over the past years to help researchers understand how environmental and neighborhood-level factors contribute to health disparities (34, 35). Since these indices rely on easily accessible databases and simple demographic forms from patients, it can be a useful tool to obtain additional factors that could impact health outcomes. However, there are some notable limitations. Common issues reported with these measures include bias for certain populations, misclassification bias (for racial/ethnic groups) and poor data quality (36). To remedy these factors, it has been recommended that multiple sources are included, using appropriate software tools and exercising caution when finding addresses that may be variable or unknown (28). Despite these challenges, there remains a critical need to examine SDH within this population to not only better understand disparities but to support policy changes that impact the most vulnerable populations. Through this research, we can begin to recognize and integrate social factors that influence health-related behaviors and health status that will ultimately develop more effective treatment plans for children with TBI. Clinically, we can also use this information to both assess and address social needs through appropriate referrals at the onset of the injury when they enter our hospital system to ensure adequate support. Ultimately, clinicians and researchers can work together to build a more equitable healthcare system that enables better health outcomes for all children.

The current study is not without limitations. First, the small sample size did not allow adequate statistical analysis. Categorization by racial and ethnic category and SDH also resulted in small sample sizes, which underpowered our ability to observe results. Due to these small sample sizes, we also chose to focus on the largest sub groups, thus it is not representative of all race and ethnicity categories, including those who identified as 2 or more groups. Similarly with functional outcomes, the patient cohort was smaller due to the high study drop-out rate and due to only following up with patients with moderate to severe brain injury. Additionally, this study only examined outcomes at 6 months post injury. It is possible, and even likely, that these social and environmental factors may not be as strongly related to short term outcomes but are more impactful to long-term functional outcomes. Exploring other functional outcomes, including neurocognitive functioning, behavioral factors, and emotional status, may provide a more comprehensive understanding of outcomes and should be planned in future studies. Additionally, our null findings with regard to race and mortality may be due to the imbalance of racial categories in our sample the fact that mortality was a rare outcome and challenging to model, or other unique aspects of the local context in which this study took place including a larger proportion of younger patients.

Last, we are aware that there may be racial bias in the sample, due to medical mistrust in minority communities and the hesitancy to join research studies leading to less minority participants (37). The majority white sample is not representative of pediatric TBI injuries at our hospital and may over represent those participating families. It is essential to include all racial/ethnic groups within future studies and try to provide comprehensive studies, such as ones with mixed method analysis (30). These methods would help to validate geocoded data while also obtaining greater insight into the long-term outcomes following TBI.

In conclusion, our study found that racial disparities in pediatric TBI do exist as measured by the severity of injury, mechanism of injury and social variables. However, there were no differences in the functional outcomes as measured by mortality and GOS-E scores. Based on these findings, future work should utilize the potential of geocoding to understand the effects of SDH on pediatric TBI outcomes, while also exploring more long-term outcomes and quality of life measures to get a more comprehensive understanding of factors that affect pediatric TBI.

## Data availability statement

The raw data supporting the conclusions of this article will be made available by the authors, without undue reservation.

## Ethics statement

The studies involving humans were approved by the Children's Healthcare of Atlanta Institutional Review Board. The studies were conducted in accordance with the local legislation and institutional requirements. Written informed consent for participation in this study was provided by the participants' legal guardians/next of kin.

## Author contributions

KP: Conceptualization, Data curation, Writing – original draft, Writing – review & editing. MM: Conceptualization, Data curation, Writing – original draft, Writing – review & editing, Methodology. GB: Writing – review & editing. SG: Writing – review & editing, Formal analysis. LJ: Formal analysis, Writing – review & editing, Visualization. HM: Writing – review & editing. AR: Writing – review & editing, Conceptualization, Methodology, Project administration. LB: Writing – review & editing, Conceptualization, Data curation, Investigation, Methodology, Project administration, Supervision, Writing – original draft.
